# Green Synthesis of Silver Nanoparticles Coated by Water Soluble Chitosan and Its Potency as Non-Alcoholic Hand Sanitizer Formulation

**DOI:** 10.3390/ma15134641

**Published:** 2022-07-01

**Authors:** Ika O. Wulandari, Baiq E. Pebriatin, Vita Valiana, Saprizal Hadisaputra, Agus D. Ananto, Akhmad Sabarudin

**Affiliations:** 1 Chemistry Department, Faculty of Mathematics and Natural Science, Universitas Brawijaya, Malang 65145, Indonesia; baiqemalia@student.ub.ac.id (B.E.P.); vita2404@student.ub.ac.id (V.V.); 2 Educational Chemistry Department, Faculty of Teacher Training and Education, University of Mataram, Mataram 83125, Indonesia; rizal@unram.ac.id; 3 Pharmacy Department, Faculty of Medicine, University of Mataram, Mataram 83125, Indonesia; agus_da@unram.ac.id

**Keywords:** silver, nanoparticle, hand sanitizer, green synthesis, chitosan

## Abstract

The synthesis of silver nanoparticles using plant extracts, widely known as a green synthesis method, has been extensively studied. Nanoparticles produced through this method have applications as antibacterial agents. Bacterial and viral infection can be prevented by use of antibacterial agents such as soap, disinfectants, and hand sanitizer. Silver nanoparticles represent promising hand sanitizer ingredients due to their antibacterial activity and can enable reduced use of alcohol and triclosan. This study employed silver nanoparticles synthesized using *Kepok* banana peel extract (*Musa paradisiaca* L.). Nanoparticle effectiveness as a hand sanitizer can be enhanced by coating with a biocompatible polymer such as chitosan. The characterization of silver nanoparticles was conducted using UV-Vis, with an obtained peak at 434.5 nm. SEM-EDX analysis indicated nanoparticles with a spherical morphology. Silver nanoparticles coated with chitosan were characterized through FTIR to verify the attached functional groups. Gel hand sanitizers were produced using silver nanoparticles coated with different chitosan concentrations. Several tests were undertaken to determine the gel characteristics, including pH, syneresis, and antibacterial activity. Syneresis leads to unstable gels, but was found to be inhibited by adding chitosan at a concentration of 2%. Antibacterial activity was found to increase with increase in chitosan concentration.

## 1. Introduction

The emergence of new pathogens, including the coronavirus responsible for the current COVID-19 pandemic, has presented new challenges for the protection of public health worldwide. This global phenomenon has stimulated the most widely used strategy to deal with potential pathogens, which is prevention of infection by attention to hand hygiene. The use of hand sanitizers, without water and soap, in the form of foam, gel, and liquid, is a practical approach to sanitization that is readily available and widely recognized in the community. The three forms of hand sanitizer vary in their effectiveness in killing bacteria and viruses due to the different mechanical friction methods they employ to physically remove pathogens [1]. It has been found that hand sanitizer in gel form is considered more beneficial than foam or liquid sanitizers, based on adherence and effectiveness, due to properties such as the ability to create a protective layer at the site of application, longer protection time on the skin, an enhanced moisturizing sensation, adherence to the skin, and higher retention time [2]. Currently, the most effective hand sanitizer products are those with an alcohol content of 62–95%, because of the effect of alcohol in denaturing microbial proteins and inactivating viruses. However, continuous or excessive usage of hand sanitizers containing alcohol with this formulation poses several challenges and concerns due to negative effects such as burning and damage to the skin, especially for sensitive types of skin [3]. Therefore, the development of non-alcoholic active ingredients as safe antibacterial and antiviral agents is necessary.

There have been a wide range of applications of nanotechnology in biomedical science, including the use of various oxides such as those of copper (Cu) [4], zinc (Zn) [5], gold (Au), and silver (Ag) [6], as well as titanium dioxide (TiO) [7], and magnesium oxide (MgO) [8]. Silver is used in metal nanoparticles for biomedical applications because of their non-toxic properties and effective antibacterial activity. Anamala and Nallamuthu [9] reported that silver nanoparticles were able to kill 650 types of microorganism.

Furthermore, silver (Ag) nanoparticles have been widely studied due to their antifungal and antiviral properties. Silver in the form of nanoparticles has a large surface-area-to-volume ratio, enabling penetration of bacterial cell walls, changing the structure of cell membranes, and even killing of targeted cells. Silver nanoparticles work by releasing silver ions, increasing cell membrane permeability, producing reactive oxygen species, and interfering with deoxyribonucleic acid replication. The safety of silver nanoparticles has been widely recognized, with no reports of systemic toxicity resulting from ingestion of silver nanoparticles [10].

According to FDA regulations, silver nanoparticles must comply with new drug application standards (NDA) with respect to their composition, effectiveness, labeling, manufacturing methods, and safety for commercialization. The FDA explicitly allows the use of silver nanoparticles in the biomedical field where they are shown to be safe and biocompatible, and where they meet appropriate quality standards in the manufacturing process. Recently, several silver nanoparticle products have been used in fabrics, cosmetics, storage containers, and medicine [11], reflecting the biocompatibility of silver nanoparticles for application to the skin.

Lu et al. [12] investigated the toxicity to the skin of silver nanoparticles coated with biocompatible polymers with light exposure. It was found that silver nanoparticles in colloidal form which were exposed to sunlight for one to three weeks did not show a toxic effect on keratinocyte cells. However, silver nitrate samples caused up to 98% cell death within one week, even with half the dose of silver nanoparticles. It was concluded that colloidal silver nanoparticles are stable and safe for use on the skin.

Physical, chemical, or biological methods for the synthesis of nanoparticles can be classified in terms of two main types of approach: (1) the top-down approach, which breaks down larger structures into smaller parts, and (2) the bottom-up approach, which involves the synthesis of materials at the atomic level to create larger nanostructures [13]. Various metal nanoparticles have previously been fabricated using top-down approaches, such as mechanical milling [14,15]), etching [16], laser ablation [17], sputtering [18], and electro-blasting [19]. Bottom-up approaches to the synthesis of nanostructures have included the synthesis of supercritical fluids [20], the use of templates [21], sol-gel processes [22], laser pyrolysis [23], molecular condensation [24], chemical reduction [25,26] and green synthesis [27,28,29].

Among these methods, biological approaches to the synthesis of nanoparticles using plant extracts, also referred to as green synthesis methods, have been more widely used than physical or chemical methods. Such biological methods are considered to have more advantages in terms of being easier to use, more economical, and more environmentally friendly. The constituents of some plants can act as metal bioreductors and capping agents for the synthesis of silver nanoparticles. Several studies have reported successful silver nanoparticle synthesis by application of bioreductors derived from plant extracts, such as *Clitoria ternatea* and *Solanum nigrum* leaf extract [30], neem leaf extract (*Azadirachta indica*) [31], Moringa leaf extract (*Moringa oleifera*) [32], teak (*Tectona grandis*) seed extract [33], and *Hagenia abyssinica* leaf extract [34]. Another plant that can act as a bioreductor for silver nanoparticles is the *Kepok* banana (*Musa paradisiaca* L.), the peel extract of which contains several polyol and heterocyclic compounds. The *Kepok* banana is widely utilized for various food manufacturing purposes. However, its peel is usually not reusable and is thrown away as waste, creating an opportunity for its use as a source of reducing agents for silver nanoparticles [35]. However, little research related to the synthesis of silver nanoparticles from *Kepok* banana peel as an alternative material for hand sanitizers has been undertaken. Thus, further research is needed for the development of non-alcohol-based hand-sanitizer products.

Ashmore et al. (2018) reported that silver nanoparticles are modifiable by coating with polymers such as polyethylene glycol (PEG), polyvinyl alcohol (PVA), citrate and sodium dodecyl sulfate [36]. Such polymers can act as stabilizing agents, preventing the aggregation of particles and promoting the interaction between silver nanoparticles and bacteria cells, inducing biological activity. Chitosan is a type of biocompatible polymer, widely applied in biomedical applications, which exhibits antibacterial activity. Chitosan has the potential to be used as a capping agent on the surface of silver nanoparticles to increase biocompatibility and the stability of nanoparticles to prevent agglomeration [37]. In this study, a non-alcoholic and non-triclosan hand sanitizer formulation was developed with silver nanoparticles modified with biocompatible molecules in the form of chitosan. The research involved the synthesis of silver nanoparticles using *Kepok* banana peel extract and assessment of the effect of chitosan concentration on the characteristics of hand sanitizer gels produced using silver nanoparticles, including their antibacterial activity.

## 2. Materials and Methods

### 2.1. Materials and Instrumentation

The following materials and chemicals were obtained from Merck: silver nitrate (AgNO_3_, Merck Pte.Ltd., Singapore), low molecular weight chitosan (deacetylation degree 75–85%, Merck Pte.Ltd., Singapore), acetone (C_3_H_6_O, 99.5%, Merck Pte.Ltd. Singapore), thickening agent carbopol 940 (Merck Pte.Ltd., Singapore), methyl paraben (C_8_H_8_O_3_, Merck Pte.Ltd., Singapore), acetic acid glacial (CH_3_COOH, 99%, Merck Pte.Ltd. Singapore), sodium hydroxide (NaOH, Merck Pte.Ltd., Singapore), and hydrogen peroxide (H_2_O_2_, Merck Pte.Ltd., Singapore). In addition, Kepok banana (*Musa paradisiaca* L.) peel extract, as a bioreductor, was collected from Malang, East Java, Indonesia. UV-visible spectroscopy (UV-Vis, Shimadzu 1601 Series), Fourier transform infrared spectroscopy (FTIR, Shimadzu 8400s), dynamic light scattering (DLS, Malvern Zetasizer), scanning electron microscopy-energy dispersive X-ray spectroscopy (SEM-EDX, Hitachi TM 3000), and X-ray diffraction (XRD, PANalytical Japan) were used for the characterization of materials.

### 2.2. Methodology

#### 2.2.1. Preparation of Kepok Banana (*Musa paradisiaca* L.) Peel Extract

Preparation of *Kepok* banana peel extract was conducted following the procedure performed by Ibrahim [35], with several modifications. Firstly, *Kepok* banana peel was cleaned with running water before being dried. The banana skin was then peeled and cut into small pieces. A quantity of 50 mL of aquabidest was placed into a 250 mL beaker and heated to boiling point. Next, 50 g of *Kepok* banana peel was boiled in distilled water for 30 min at 85 °C. The boiled water filtrate was then separated from the peels using a cheesecloth. After the separation process, acetone was added to the filtrate in a 1:1 ratio and the filtrate centrifuged at 1000 rpm for five minutes. The resulting precipitates were then filtered using Whatman paper No. 1 and were suspended in distilled water in a ratio of 1:30 mL. Lastly, the extract was stored at 4 °C in a refrigerator for further study.

#### 2.2.2. Synthesis and Characterization of Chitosan-Modified Silver Nanoparticles

Prior to the synthesis of silver nanoparticles, AgNO_3_ solution was prepared by dissolving 0.03 g of AgNO_3_ in 100 mL of distilled water, resulting in a 1.75 mM AgNO_3_ solution. The synthesis of silver nanoparticles with *Kepok* banana peels was carried out by mixing *Kepok* banana peel extract with AgNO_3_ solution in a 1:1 volume ratio. Afterwards, the solution was incubated in a dark room for 24 h at 30 °C. The formation of silver nanoparticles was indicated by a change in the color of the solution from a clear to a brownish-yellow. The resulting silver nanoparticles were coated utilizing water-soluble chitosan. As an independent variable in this research, silver nanoparticles were treated by coating with chitosan at different concentrations of 0.5%, 1%, and 2%. For comparison, silver nanoparticles without chitosan coating were also used for further analysis. The coating process of synthesized silver nanoparticles was performed by adding a chitosan solution with 0%, 0.5%, 1%, and 2% concentrations, which had previously been optimized through depolymerization using a solution of H_2_O_2_, 10% of sodium hydroxide, and acetic acid. Silver nanoparticles, with the previous addition of chitosan, were stirred using a magnetic stirrer for 2 h, centrifuged, and further washed with acetone. Before being characterized, the nanoparticles were dried using a freeze-drying process. The silver nanoparticles were then characterized by FTIR to determine the nature of the functional groups before and after coating with chitosan. The silver nanoparticles were also characterized by XRD and SEM-EDX to confirm the formation of silver nanoparticles and to determine their crystal structure and morphology. Determination of the particle size distribution of silver nanoparticles, before and after coating with chitosan, was performed using DLS.

#### 2.2.3. Preparation of Hand Sanitizer Gel

A quantity of 0.5 g of carbopol 940 was sprinkled over 20 mL of distilled water in a mortar and stirred until a gel mass was formed. A quantity of 0.1 g of methyl paraben was weighed and dissolved in 5 mL of distilled water in a mortar and stirred until the mixture was homogeneous. A quantity of 4 mL of glycerin was added and stirred until the mixture was homogeneous. Quantities of 5 mL of silver nanoparticles with or without chitosan coating were added to the mixtures and then stirred until dissolved. Then, 20 mL of distilled water was added, mixed until homogeneous and crushed until a gel was formed. Through this procedure, four different compositions of gel product containing silver nanoparticles without chitosan, and silver nanoparticles coated by chitosan with three different compositions of chitosan (0.5; 1; and 2%) were obtained. The resulting gels were then placed in a container to be further tested.

#### 2.2.4. pH and Syneresis Test of Gel

The acidity of the gel hand sanitizer was assessed to determine the compatibility of the gel with human skin. The pH measurement was performed by dipping the pH meter into the gel product at room temperature. A syneresis test was also performed. The syneresis test was conducted by observing the weight of the gel before and after being placed in storage at two different temperatures of 5 °C and 40 °C for 140 h.

#### 2.2.5. Antibacterial Activity Test

An antibacterial activity test was performed using the well method to determine the inhibition zone of each hand sanitizer gel product. The first step was performed to produce a sterile nutrient medium which was then chilled to a temperature of 40–45°. Afterwards, the solid media were smeared with cultures of gram-positive (*Staphylococcus aureus*) and gram-negative (*Eschericia coli*) bacteria utilizing sterile cotton buds in different cups. After smearing with bacterial cultures, wells were made in the compacted *agar* medium using an iron perforator or a clamp. The hand sanitizer gel sample was then inserted into the wellbore, which was cleaned to remove gram-positive and gram-negative bacteria. A commercial hand sanitizer gel containing 70% alcohol and 0.2% phenoxyethanol incubated at 37 °C for 24 h was used as a positive control. The zone of inhibition of each sample was calculated by measuring the diameter of the clear area using a caliper.

#### 2.2.6. Molecular Docking Method

Molecular docking was conducted to compare the experimental results with in silico theoretical results utilizing ChemAxon’s marvinSketch 5.2.6, YASARA Bioscience’s YASARA View 19.12.14, and ChemAxon’s PLANT. There were two types of ligands: standard ligands and test ligands. SB 3gr6 receptors, generated from the Protein Data Bank were used in this study [38]. A three-step docking technique was used. The first step involved synthesis of the target protein and native ligands. The second step involved validation of the docking protocol, and the third step involved docking of the test ligand. YASARA View was employed to prepare the native ligands and target proteins by removing the native ligand of the protein. By re-docking the native ligand to its protein using YASARA View, the docking protocol validation enabled an RMSD (root median square deviation) value to be obtained. The validation of the docking process was considered accurate if the RMSD value was less than 2 [39]. PLANT was employed to type the commands for test ligand docking in cmd.exe. SB 3gr6 active sites were coupled with the test ligand. PLANT was used to analyze the commands to determine the best docking score. Combining of docking techniques was performed to establish a synergistic relationship between the chemicals and to bind ligands to each target protein with sufficient stability. The compounds were arranged based on their average docking score to create the docking protocol combination. The compounds with the highest docking scores and the target protein were further saved in a file called protein.mol2 and docked to the compounds with the lowest docking scores.

## 3. Results and Discussion

### 3.1. Synthesis of Silver Nanoparticles with Kepok Banana Peel Extract (Musa paradisiaca L.)

**Figure 1:**
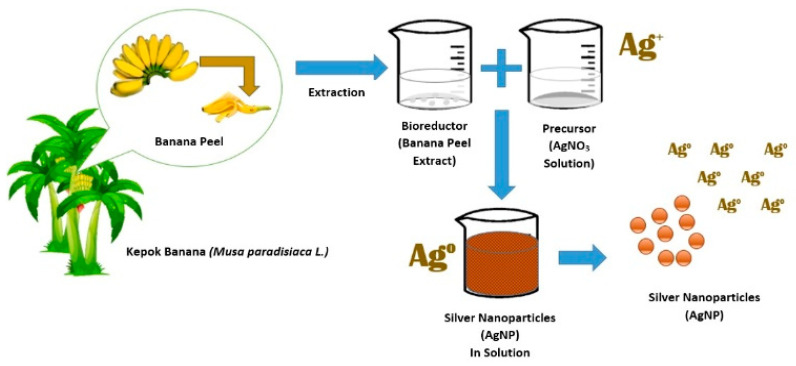
Ilustration of silver nanoparticle formation using banana peel extract as bioreductor.

Green synthesis of silver (Ag) nanoparticles in an aqueous solution was conducted by mixing an AgNO_3_ precursor with *Kepok* banana peel extract as a bioreductor containing active compounds of flavonoids, alkaloids, tannins, saponins, and triterpenoids [40]. Mixing the precursor solution with banana peel extract reduced the Ag ions to produce silver nanoparticles (Ag). In general, a biosynthesis approach using plant extracts is more widely employed than chemical or physical methods. The biosynthesis process used to produce silver nanoparticles using *Kepok* banana peel extract is illustrated in Figure 1.

All parts of plants, including the leaves, fruits, roots, seeds, and stems contain biomolecules, such as enzymes, alkaloids, flavonoid, polysaccharides, tannins, terpenoids, phenols, and vitamins which serve not only as agents to reduce Ag^+^ ions to Ag^0^ for the synthesis of silver nanoparticles but also as capping agents for the surface of nanoparticles produced.

**Figure 2:**
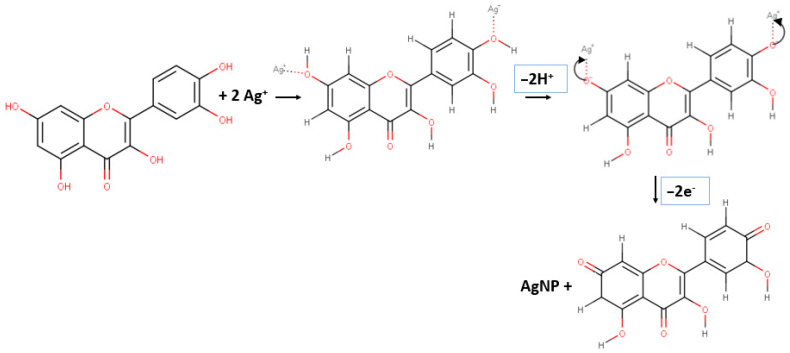
A possible reaction mechanism for silver nanoparticles in the presence of plant extract of *Kepok* (*Musa paradisiaca* L.) banana peel.

The hydroxyl group attached to the carbon atom of the aromatic ring enabled reduction of silver ions to produce silver nanoparticles. Due to a lower dissociation energy than for other OH groups, the catechol OH group plays an important role in reducing metal ions. One molecule of flavonoid and polyol compounds will produce two protons per catechol, so that one molecule can reduce two silver ions [41]. The proposed reaction mechanism for the formation of silver nanoparticles using *Kepok* banana peel extract is illustrated in Figure 2.

**Figure 3:**
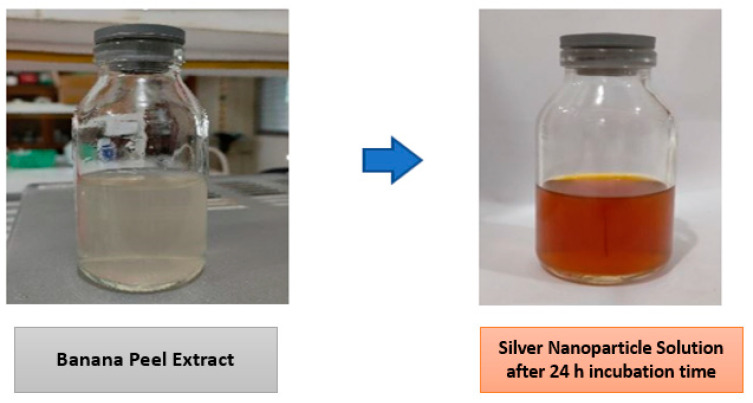
The color change of silver nitrate solution after adding banana peel extract within 24 h of incubation.

The formation of Ag nanoparticles was characterized by a change in the color of the solution. Initially, the obtained AgNO_3_ solution was clear. However, after being mixed with *Kepok* banana peel extract, within 24 h of incubation time the color of the solution turned brownish yellow, as shown in Figure 3.

Silver nanoparticles that had been successfully synthesized were then modified by adding a water-soluble chitosan solution with different concentrations of 0.5, 1 and 2% chitosan. In this study, chitosan was used as a stabilizing agent on the surface of the silver nanoparticles to prevent agglomeration. In our previous study, it was found that chitosan added as a coating agent on the surface of nanoparticles was able to prevent agglomeration in a sample of iron oxide nanoparticles by decreasing the size of iron oxide clusters [42].

Similarly, Cinteza et al. [43] reported that chitosan acts as a stabilizing agent for silver nanoparticles preventing nanoparticle agglomeration by breaking particle clusters into smaller sizes. Chitosan coating of the surface of silver nanoparticles was able to prevent the formation of aggregates with other silver nanoparticles. In this study, aggregation only occurred if there was no addition of a stabilizing agent (such as chitosan) to the nanoparticle samples.

In principle, bare metal nanoparticles will readily agglomerate due to van der Waals interactions that occur on the surface. Phan et al. [44] stated that chitosan was an excellent stabilizing agent for metal nanoparticles. Chitosan acts as a steric barrier by covering the metal surface with a positive charge. The use of chitosan as a stabilizing agent has been demonstrated in the synthesis of gold nanoparticles using chitosan and citric acid reagents. The presence of chitosan stabilized the nanoparticles, thereby preventing agglomeration.

Chitosan was dissolved in water to form a homogeneous hand sanitizer gel formulation. Silver nanoparticles without modification were used as a control in the observations. The formation of silver nanoparticles was observed using a UV-Vis spectrophotometer and SEM-EDX for the purpose of characterization. In addition, samples of chitosan modified silver nanoparticles were observed for functional group characterization using FTIR. The gel stability and antibacterial properties of hand sanitizer gel formulated using both modified and unmodified silver nanoparticles were tested.

### 3.2. Characterization of Silver Nanoparticles Using UV-Vis Spectrophotometer

**Figure 4:**
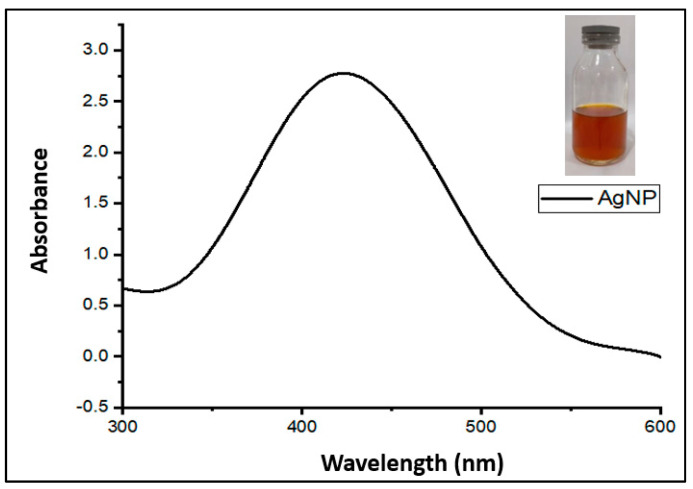
UV-Vis absorption spectrum of silver nanoparticles.

Characterization of silver nanoparticles with a UV-Vis spectrophotometer was conducted to observe the characteristic absorption peaks or surface plasmon resonance of the silver particles in the sample using distilled water as a blank solution. Sawalha et al. [45] and Razy et al. [46] reported that the UV-Vis instrument is an essential instrument to obtain information related to the SPR peaks in silver nanoparticle formation. To complement UV-Vis, nanoparticles can be characterized by EDX, XRD, and FTIR. The results of UV-Vis characterization showed that the solution containing silver nanoparticles produced peaks with a maximum wavelength around 400 nm. The UV-Vis spectrum of the silver nanoparticle samples produced in this study is illustrated in Figure 4.

The characterization results indicated that the absorbance peak, or surface plasmon resonance (SPR), appeared as a single peak at a maximum wavelength of 434.5 nm. According to a previous study, it was found that silver nanoparticle absorption peaks occurred especially in the wavelength range of 390 to 470 nm [47]. According to Jyoti et al. [48], SPR peaks in the 410–450 nm wavelength region indicate nanoparticle materials with a more specific spherical shape. However, to confirm hypotheses regarding the shape or morphology of nanoparticles, further testing is required using other instrumentation, such as SEM-EDX.

In the formation of silver nanoparticles, several factors affect the size and shape of the resulting nanoparticles, including the effect of the extract concentration and incubation time. If the concentration of the extract is lower than that of the precursor, it will result in the formation of nanoparticles with a larger size due to a slower rate of nuclei formation. However, if the concentration of the extract is too high, it will quickly increase the reaction rate and cause the nucleus to grow in a particular direction, producing nanoparticles in the form of rods [49]. In addition, the incubation time affects the formation of silver nanoparticles, with longer incubation time causing the formation of silver nanoparticles in larger quantities, reflected in a UV-Vis absorption peak profile showing absorbance values of high intensity at the maximum wavelength of silver nanoparticles.

### 3.3. Morphology and Elemental Analysis of Silver Nanoparticles Using Scanning Electron Microscope Energy Dispersive X-ray Spectroscopy

**Figure 5:**
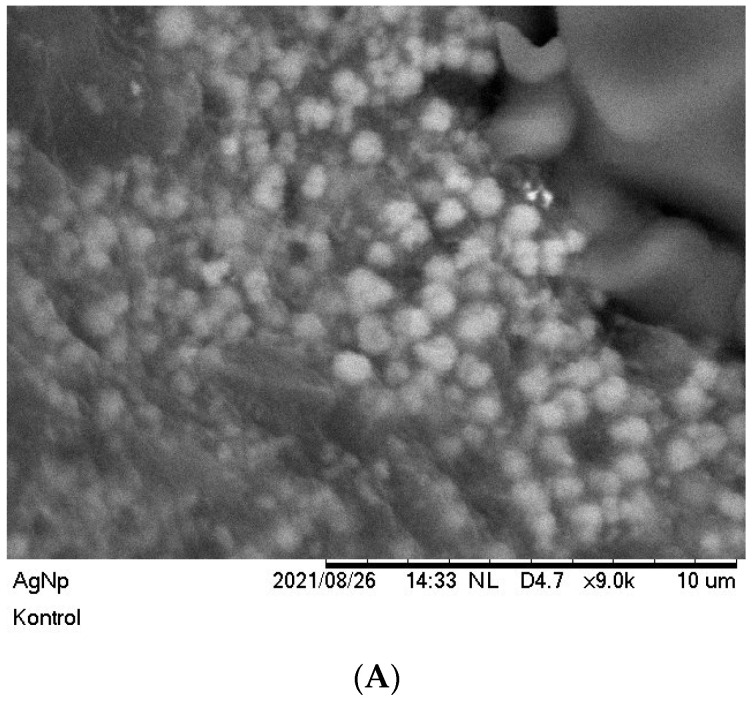

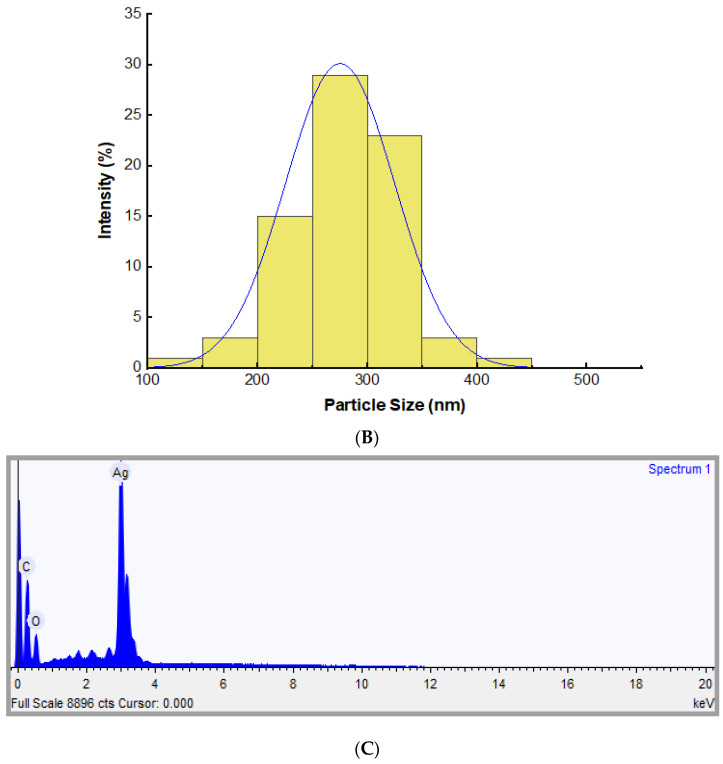
(**A**) Scanning electron microscope (SEM) image of silver nanoparticles with 9000× multiplication; (**B**) Particle size distribution of silver nanoparticles; (**C**) EDX spectra of silver nanoparticles.

A characterization process was conducted using an SEM (scanning electron microscope) to determine the morphology of the silver nanoparticles. The results indicated that the silver nanoparticles were spherical with an average particle size in the range of 100–300 nm. Previous results indicated that the maximum absorbance measured by UV-Vis spectrophotometer was around 410–450 nm, reflecting the spherical morphology of nanoparticles [48]. Additionally, a small portion of the nanoparticles underwent clustering, generating a larger size of nanoparticles, due to the large surface energy of nanoparticles and the length of storage prior to the characterization process. The result of the SEM-EDX characterization is illustrated in Figure 5.

The particle size distribution was determined by processing the SEM data using a combination of Visio and Origin software. Visio software was employed to measure the size of each single particle matched to the actual scale of particles from the SEM images. The data was then transferred into Origin software to calculate the size distribution of the silver nanoparticles. The size distribution of silver nanoparticles obtained is illustrated Figure 5B.

The EDX pattern of silver nanoparticles is depicted in Figure 5C. According to the EDX pattern, the existence of Ag nanoparticles was identified at around 3 keV which is consistent with the results obtained by Jain et al. [41]. Moreover, a prior study conducted by Kgatshe et al. [50] suggested that absorption peaks below 5 keV indicated the existence of pure silver metal ions. However, peaks corresponding to the presence of carbon and oxygen were also observed in the EDX spectra attributed to the presence of the capping agent originating from the *Kepok* banana peel extract. The EDX spectra confirmed that the nanoparticles contained about 56% silver.

### 3.4. Characterization of Silver Nanoparticles Using X-ray Diffraction

**Figure 6:**
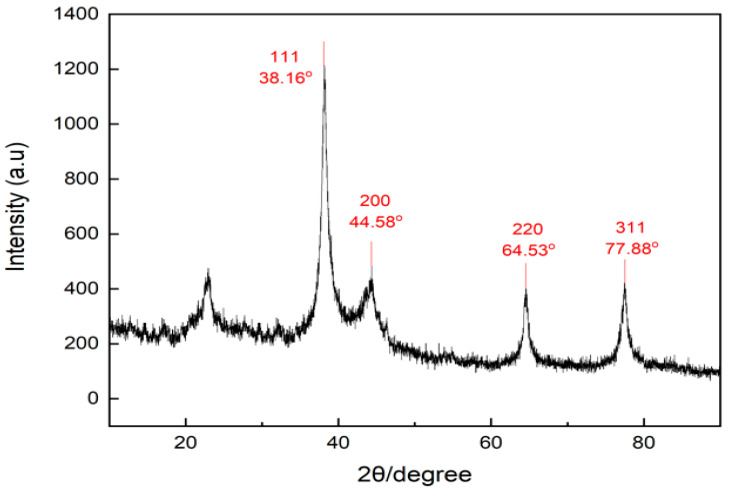
X-ray diffraction pattern of silver nanoparticles synthesized using *Kepok* banana peel extract.

The crystal structure of the silver nanoparticles was obtained using an X-ray diffraction (XRD) technique. The XRD pattern obtained is shown in Figure 6. The diffractogram of silver nanoparticles synthesized by *Kepok* banana peel extract showed an intense peak at 2 theta values of 38.16°; 44.58°; 64.53°; and 77.88°, which correspond to the standard Bragg reflections (111), (200), (220), and (311) of a face-centered cubic lattice

The additional peak observed with a lower intensity that appeared at 2 theta of 22° was due to the presence of the capping agent from the *Kepok* banana peel extract. The X-ray diffraction pattern also confirmed the formation of silver nanoparticles, consistent with earlier reports [51,52,53].

### 3.5. Functional Group Characterization of Nanoparticle Samples by Employing Fourier Transform InfraRed Spectroscopy

**Figure 7:**
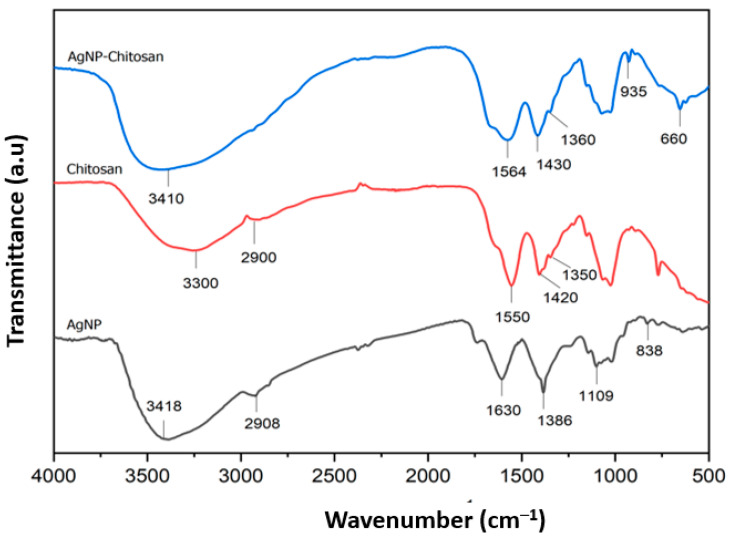
FTIR spectra of silver nanoparticles (black), chitosan (red), and silver nanoparticles coated with chitosan (AgNP-chitosan) (blue).

FTIR analysis provided details of the characteristics of the presented surface structure and of the functional groups involved in the reduction of Ag ions and of possible interactions between chitosan and silver nanoparticles. The FTIR spectra for silver nanoparticles, with and without chitosan coating, are illustrated in Figure 7. The absorption band for chitosan is depicted at wave numbers of around 3300 cm^−1^ and 2900 cm^−1^, indicating amide A and amide B bands, in which amide A bands appeared due to the presence of O-H alcohol groups or N-H amines. Amide B bands appeared mainly due to the strain vibration of the aliphatic –CH bond. The absorption bands at 1550 cm^−1^, 1420 cm^−1^, and 1350 cm^−1^ were attributed to C=O strain vibration (Amide I), NH bending vibration (Amide II), and CH_2_ wobble vibration (Amide III), respectively [54].

Meanwhile, the absorption band for silver nanoparticles coated with chitosan (AgNP-chitosan) indicated a slight change associated with chitosan. The absorption band for AgNP-chitosan observed at 3410 cm^−1^ was attributed to the amide bond. The hypochromic shift associated with this peak might have been due to the interaction of primary amino and amide groups of the chitosan and silver nanoparticles. The existence of bands at 1564 cm^−1^, 1430 cm^−1,^ and 1360 cm^−1^ confirmed the vibration of organic substances. These bands also indicated the binding of silver nanoparticles with chitosan as a stabilizing agent [13]. Based on previous research conducted by Hajj et al. [55], lower peak intensity at these bands occurs due to the interaction of Ag, O, and N atoms of amide groups when compared to chitosan spectra. These main bands for the AgNP-chitosan composite indicated the formation of coordinate bonds between amino and hydroxyl groups of chitosan and silver nanoparticles.

Furthermore, based on the results of the FTIR spectra of silver nanoparticles without chitosan coating, it was apparent that there was a characteristic peak at 3418 cm^−1^ of lower intensity compared to the chitosan and AgNP-chitosan spectra, indicating stretching vibrations of O-H or N-H groups. The presence of these phenolic and amine groups was due to the utilization of *Kepok* banana peel extract as a bioreductor for the production of silver nanoparticles (even wihout chitosan coating). This O-H group is a component of the phenolic functional group derived from the plant extract. In addition, Saha et al. [56] reported the presence of a shoulder peak that appears at a wavenumber lower than 3000 cm^−1^, indicating the presence of O-H and N-H bonds from the extract on the surface of silver nanoparticles. Moreover, the observed band at 1683 cm^−1^ indicates the presence of C=O or C=N groups derived from *Kepok* banana peel extract. The absorption band for the carbonyl group indicates the presence of flovonone or terpenoid compounds adsorbed on the surface of the nanoparticles.

### 3.6. Characterization of Particle Size Distribution of Silver Nanoparticles Coated by Chitosan Using Dynamic Light Scattering

**Figure 8:**
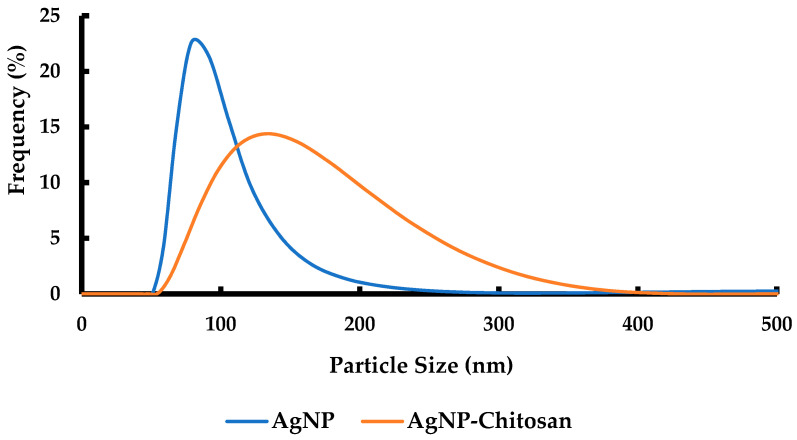
Particle size distribution of dynamic light scattering (DLS) for silver nanoparticles (AgNP) and silver nanoparticles coated by chitosan (AgNP-chitosan).

The particle size distribution of silver nanoparticles (AgNP), before and after coating by chitosan polymer, was characterized using dynamic light scattering, as presented in Figure 8. The particle size distribution varied from 51 to 255 nm, and 55 to 371 nm for silver and silver-coated chitosan, respectively. In this study, the particle size distribution of silver nanoparticles obtained by DLS was slightly different for the SEM image. This was probably because the SEM only detected the surface morphology of the nanoparticles. The smaller particles tended to form clusters after the drying process (observed by SEM). However, these were distributed in the aqueous medium (as determined by DLS).

According to the results, the silver nanoparticles coated by chitosan had a wide range of size distribution due to the size of chitosan molecules and the number of chitosan layers on the surface of the nanoparticles. However, the smallest particle size of silver nanoparticles coated with chitosan was 55 nm. The smallest particle size of the silver nanoparticles without chitosan was 51 nm. Therefore, the values for these particles were slightly different. The average particle size of the chitosan-coated silver nanoparticles was 133 nm, which was smaller than that of the particle sizes of the silver nanoparticles obtained using SEM images. This implies that the presence of the chitosan polymer collapsed the silver nanoparticle clusters to form smaller particle sizes resulting in reduced agglomeration.

### 3.7. Visual Characterization of Gel Hand Sanitizer

**Figure 9:**
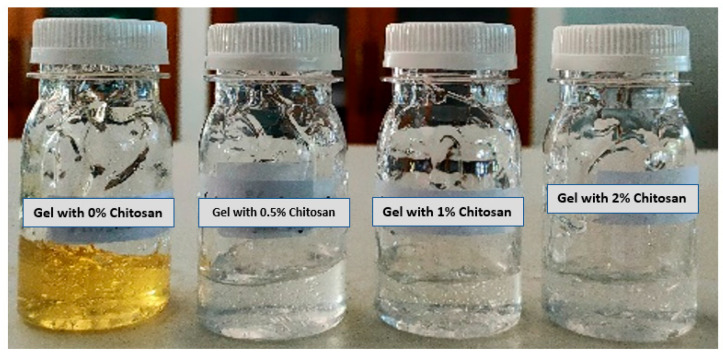
Hand sanitizer gel with different formulations containing silver nanoparticles and silver nanoparticles coated by chitosan with different chitosan concentrations.

Visual quality inspection indicated that the resulting hand sanitizer formulation possessed good characteristics, with respect to gel texture and homogeneity, with a clear golden yellow color for gels containing silver nanoparticles without chitosan coating, and a transparent color for gels containing nanoparticles with chitosan coating. Furthermore, the gels were found to be freshly scented. The hand sanitizer gels with different formulations are illustrated in Figure 9.

The color change from yellow to transparent was due to the presence of silver nanoparticles dispersed in the chitosan matrix [57]. The color change generally depends on two factors, including the amount of Ag and the average particle size. This phenomenon also indicates that silver nanoparticles undergo declustering with the addition of chitosan polymer. Therefore, the average size of the single silver particles was reduced when combined with the chitosan polymer to produce an optically transparent gel. This optical color change has also been observed during the formation of nanocomposite and hydrogel films with silver-chitosan nanoparticle components [58,59].

In addition, the gel was found to be easy to apply and to spread easily, without the presence of coarse particles when spread on transparent glass. The resulting gels exhibited similar characteristics to those reported in a study by Booq et al. [2] which found gel characteristics such as homogeneity, clarity, ease of application, lightness of spread, and consistent flow.

### 3.8. pH Test of Gel Hand Sanitizer

The pH value of the hand sanitizer gel was measured using a pH meter for each different formulation of hand sanitizer gel. The pH values for hand sanitizer gel, as prepared with 0%, 0.5%, 1%, and 2% chitosan, were 4.65, 4.66, 4.65 and 4.67, respectively. The acidity (pH) which is safe for the skin and conforms to the ideal standard for the pH value of the topical dosage should fall within a skin pH range of 4.0 to 7.0 to prevent inflammation and skin irritation.

It was reported that the optimal conditions for the growth of several pathogenic bacteria infecting the skin are in the neutral pH and alkaline pH range [17,18]. On the other hand, normal bacteria are more likely to remain on the skin if the pH conditions are slightly acidic. According to previous research reported by Booq et al. [2], the average pH value of the natural skin surface is less than 5.0, providing the optimal conditions for dermal biological processes, and thus the activity of antimicrobial compounds will enhance this condition. Thus, increasing the acidity level of hand sanitizer gel, even slightly, from neutral pH (pH 7) to a more acidic pH (pH 4.5–6.5) will boost its effectiveness against pathogenic bacteria [2,60,61].

### 3.9. Syneresis Test

Syneresis refers to the occurence of impulsive liquid released out of the gel, squeezing water from the inside, thereby shrinking and solidifying the gel [62]. According to Hesarinejad et al. [63], syneresis is affected by some organic compounds; a higher concentration of protein or other materials that contain amine groups reduces the potential for syneresis, since proteins and materials containing amine groups are able to absorb water to a greater degree. In this study, a decrease in syneresis was observed with higher concentrations of chitosan on the nanoparticle surface. During the investigation, the gels were stored in a refrigerator at ±5 °C and in an oven at ±40 °C for 140 h; it was found that gels with different chitosan concentrations at 5 °C storage temperature lost more weight than gels stored in 40 °C, as shown in Table 1.

The stability of nanoparticles with gel composition was also evaluated by Kopytov et al. [64], which indicated that silver nanoparticles coated with polymer (polyvinylpyrrolidone) had good stability at 5 °C when stored for 36 months. In this study, a syneresis test was also conducted within 140 h (±6 days) at extreme temperatures of 5 °C and 40 °C. The results of the syneresis test indicated that the gel-contained nanoparticles were stable at different compositions and only experienced a mass decrease of less than 3% and 2% for storage at 5 °C and 40 °C, respectively. These results indicated that the stability of silver nanoparticle aggregates with polymer as a stabilizing agent contained in gel form could be maintained for a long period.

The results shown in Table 1 indicate that gel formulation was relatively stable under those two conditions. Since the stability of the gel is indicated by low levels of syneresis, the gel with 1% chitosan was the most stable at a storage temperature of 5 °C. However, when both treatments are considered, gel with 2% chitosan concentration showed insignificant change. The same observations were reported by Kalia et al. [65] when considering the water holding capacity of chitosan and chitosan-metal nanocomposites. The addition of ZnO and CuO nanoparticles to nanocomposites significantly reduced their water holding capacity (WHC) by up to 37%. If the WHC decreases, an increase occurs in syneresis events. If syneresis increases, both the material and the gel will lose their stability. Therefore, increasing the chitosan concentration plays an important role in enhancing WHC, decreasing the syneresis effect.

### 3.10. Antibacterial Acitivity

**Figure 10:**
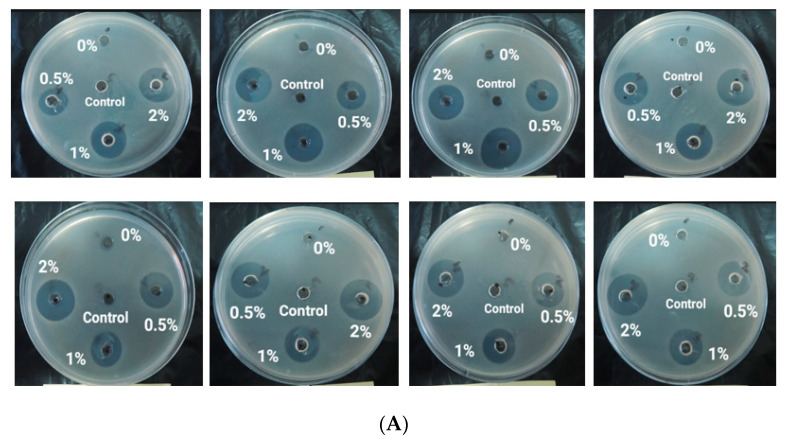

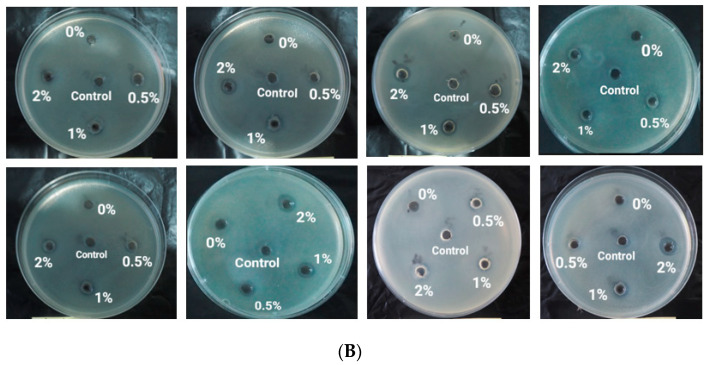
Diameter of inhibition zone of hand sanitizer gel against (**A**) *S. aureus* and (**B**) *E. coli* with eight times measurement repetition.

An antibacterial activity test was conducted using a well diffusion method against *Staphylococcus aureus* and *Eschericia coli.* Antibacterial activity is indicated by the presence of a clear zone around the well. The diameter of the clear zone around the well containing the hand sanitizer gel with different formulations was measured and compared with the positive control, a commercial hand sanitizer gel consisting of 70% alcohol and 0.2% phenoxyethanol. The results of the antibacterial activity test in this study indicated that the inhibition zone produced by chitosan-modified non-alcoholic hand sanitizer gel was larger than that for the commercial hand sanitizer gel, as shown in Figure 10, and Table 2 and Table 3.

Based on the results of the antibacterial activity test, the modified non-alcoholic hand sanitizer gels containing silver nanoparticles and chitosan exhibited a higher inhibition response compared to commercial hand sanitizer gels. However, silver nanoparticles without chitosan coating exhibited a similar clear zone diameter compared with the commercial hand sanitizer gel. This suggests that silver nanoparticles show antibacterial activity. However, antibacterial activity significantly increased compared to commercial hand sanitizer gel with the addition of chitosan. Therefore, chitosan has a synergistic effect, enhancing the antibacterial activity of silver nanoparticles in the formulation of gel hand sanitizers.

Silver nanoparticles coated with a polymer (chitosan) have also been successfully synthesized in several previous studies [52,66,67]. A similar diameter of the inhibition zone was found in the present study compared to previous research. The average diameter of the inhibition zone resulting from adding nanoparticles into bacterial strains (*S. aureus* and *E. coli*) was found to be 8–19 nm [53,68,69]. The diameter depended on the amount of chitosan and silver nanoparticles in the different samples.

According to the diameter of inhibition zone obtained in this study, modified hand sanitizer gel with chitosan had a greater impact on *S. aureus* than on *E. coli* bacteria. Similar results were obtained (Table 4) by Nithya et al. [69] and Mirda et al. [52] using a nanocomposite product, as well as by Ahmad et al. [66], using a produced hydrogel. The effect occured because *S.* *aureus* is a gram-positive bacterium with a simple, single-layered cell wall structure with low lipid content, enabling bioactive compounds to penetrate the cells. *E. coli*, however, is a gram-negative bacterium with a more complex cell structure, having a three-layer lipoprotein coat, consisting of an outer layer, a middle layer of lipopolysaccharide, and a peptidoglycan layer with high lipid content. These layers act as a barrier to antibacterial bioactive ingredients, thus hindering the penetration of the cell membrane. However, this study confirmed that hand sanitizer gels containing silver nanoparticles and chitosan achieved higher antibacterial activity against pathogenic bacteria [70] by comparison with the antibacterial activity of silver nanoparticles coated by chitosan against *S. aureus* bacterial strain observed in previous research. 

In our previous research, we also investigated the phenomenon of bacteria-killing by silver nanoparticles using FE-SEM. FE-SEM images obtained indicated that silver nanoparticles changed bacteria cell morphology. Bacterial cell death occurred due to swelling and shrinking mechanisms in the bacterial cells [51].

### 3.11. Docking Studies

This study sought to validate the experimental results against in silico theoretical results for docking studies. The protein data bank (http://www.rcsb.org./pdb, accessed on 24 January 2022) was employed to obtain the crystal structures of *Staphylococcus aureus* enoyl-acyl carrier protein reductase in complex with NADP and triclosan (PDB ID: 3gr6). Table 5 displays the docking score values obtained during the molecular docking stage of chitosan to receptors. With a spontaneous process, the highest docking score energies represent the best possible geometry of the compounds inside the protein.

**Figure 11:**
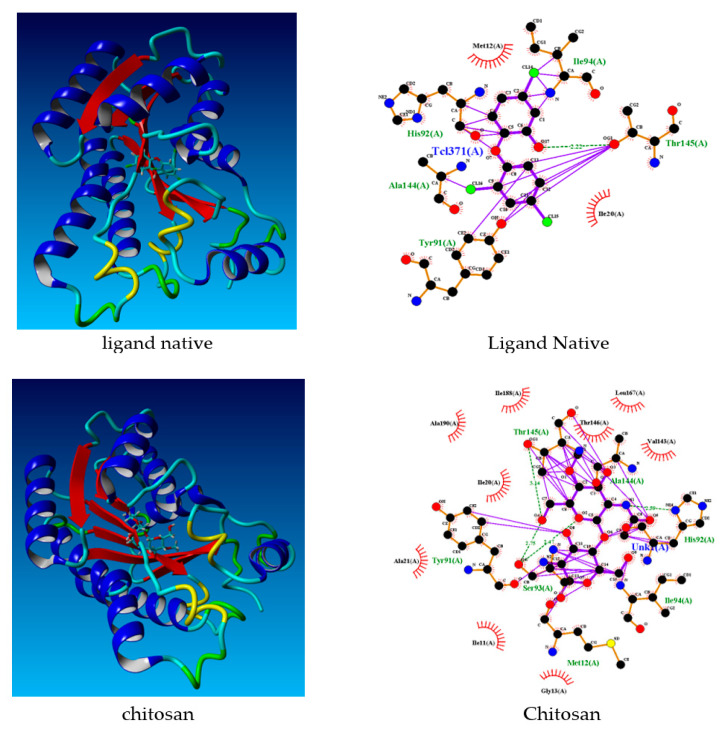

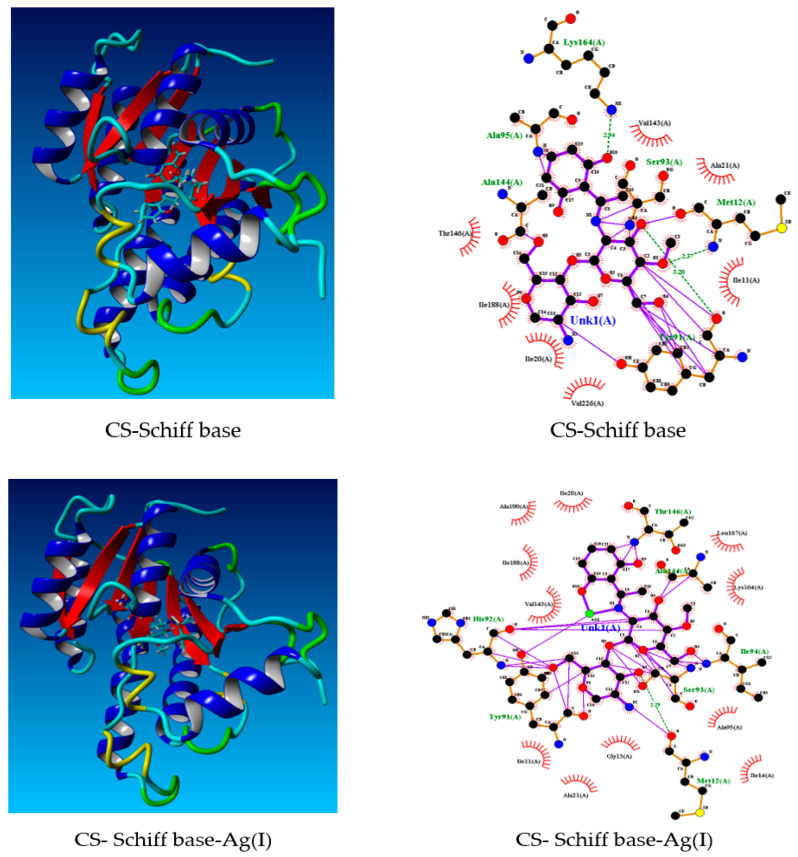
Docking conformation and bonding interactions of native ligand, chitosan, CS-Schiff base and CS-Schiff base-Ag(I).

Table 5 illustrates the values of docking scores for native ligand, chitosan, chitosan-Schiff base, and chitosan-Schiff base-Ag(I) which were: −72.8008 kcal·mol^−1^, −81.1968 kcal·mol^−1^, −85.8808 kcal·mol^−1^, and −92.4815 kcal·mol^−1^, respectively. The chitosan, chitosan-Schiff base, and chitosan-Schiff base-Ag(I) compounds showed lower binding energies than their native ligands. Table 6 depicts all the compounds’ interactions. The docking conformation and bonding interactions of ligands are depicted in Figure 11.

In sum, the findings indicated that the native ligand was more selective for 3gr6 than chitosan and chitosan-Ag complexes. In addition, the computational studies suggested that chitosan compounds were potential inhibitors in the treatment of antibacterial infections. Silver nanoparticles added to chitosan (chitosan-Schiff base-Ag(I)) had the most antibacterial potential. The results of this theoretical study strengthen the experimental findings.

## 4. Conclusions

Hand sanitizer gel is preferred as an alternative for hand hygiene. This study involved the formulation of gels using chitosan-modified silver nanoparticles as an active antimicrobial agent. The results indicated that silver nanoparticles with a spherical shape were obtained using *Kepok* banana peel extract. The nanoparticles were formulated as gel preparations with several chitosan concentrations. According to the results of pH, syneresis, and antibacterial activity tests, it can be concluded that the gel formulations produced, with or without chitosan, exhibited excellent characteristics with appropriate pH values (less than 5) (comparable to skin pH), high stability, and effectiveness as antibacterial agents. Furthermore, the presence of chitosan as a coating on the surface of silver nanoparticles acted as a stabilizing agent to prevent agglomeration, increasing the effectiveness of nanoparticles as an antibacterial agent. In summary, silver nanoparticles, produced by a green synthesis method with chitosan coating, are promising for the formulation of gel hand sanitizer products without alcohol content.

## Figures and Tables

**Table 1:** Syneresis test result at two different temperatures of 5 °C and 40 °C for different gel formulation.

Chitosan Concentration in Gel Formulation	Storage Temperature (5 °C)			Storage Temperature (40 °C)		
	Gel Mass before Storage (g)	Gel Mass after Storage (g)	Gel Mass Loss (%)	Gel Mass before Storage (g)	Gel Mass after Storage (g)	Gel Mass Loss (%)
0%	10.2885	9.994	2.87	10.3795	10.3650	0.14
0.5%	10.3863	10.2110	1.69	10.0369	9.9236	1.13
1%	10.3235	10.2021	0.32	10.6330	10.423	1.97
2%	10.3787	10.2685	1.06	10.0811	9.975	1.05

**Table 2:** Antibacterial activity of hand sanitizer gel towards *Staphylococcus aureus* bacteria.

Dose	The Diameter of Inhibition Zone (mm)								Average
	1	2	3	4	5	6	7	8	
0%	6.00	6.00	6.00	6.00	6.00	6.00	6.00	6.00	6.00
0.5%	17.11	15.37	15.49	17.06	19.01	19.02	17.25	18.17	17.31
1%	20.54	19.22	16.83	18.19	19.31	16.77	16.84	17.46	18.14
2%	20.12	19.41	17.23	19.59	22.15	18.18	18.09	20.34	19.39
Positive Control	7.08	6.81	7.03	6.74	6.66	6.59	7.45	6.78	6.89

**Table 3:** Antibacterial activity of hand sanitizer gel towards *Escherichia coli* bacteria.

Dose	The Diameter of Inhibition Zone (mm)								Average
	1	2	3	4	5	6	7	8	
0%	6.00	6.00	6.00	6.00	6.00	6.00	6.00	6.00	6.00
0.5%	7.24	8.56	8.67	9.18	7.25	8.44	6.29	8.51	8.02
1%	8.61	8.42	9.12	9.17	8.38	10.14	7.83	8.23	8.74
2%	9.29	7.91	10.45	10.09	8.87	9.85	10.04	8.37	9.36
Positive Control	6.15	6.17	7.29	7.06	7.11	7.21	6.73	6.42	6.77

**Table 4:** Comparison of antibacterial activity of silver nanoparticles coated with chitosan against *S. aureus* bacterial strain for previous and current studies.

Dose/Composition	Diameter of Inhibition Zone (mm)	Source
AgNP-Chi-Spheres (in 20% NaOH)	15.40	[52]
AgNP coated Chitosan	8.80	[66]
AgNP-Chitosan (Chitosan + 2% AgNP)	12.42	[68]
Chitosan-Ag (10 µg)	13.00	[69]
AgNP coated Chitosan (with 2% Chitosan)	19.29	Current Research

**Table 5:** The docking results of native ligand, chitosan, chitosan-Schiff base and chitosan-Schiff base Ag(I) to 3gr6.

Ligand	Docking Score against 3gr6 (kcal·mol^−1^)
Ligand native	−72.8008
Chitosan	−81.1968
CS-Schiff base	−85.8808
CS-Schiff base-Ag(I)	−92.4815

**Table 6:** The interaction of *Staphylococcus aureus* amino acids with various compounds.

	Residue	Hydrogen Bond
Native ligand	Ile94, Thr145, Tyr 91, Ala144, His92	Thr 145 (2.22 Å)
Chitosan	Thr145, Ala144, His92, Ile94, Met12, Ser93, Tyr91	His92 (2.59 Å), Thr145 (3.14 Å), Ser93 (2.47 Å) dan Ser 93 (2.75 Å)
Chitosan SB	Lys164, ser93, Met12, Tyr91, Ala144, Ala95	Met12 (2.37 Å), Tyr91 (3.20 Å), Lys164 (2.94 Å)
Chitosan SB Ag(I)	Thr146, Ala144, Ile94, Ser93, Met12, Tyr91, His92	Met12 (2.29 Å)

## Data Availability

Not applicable.
